# General Health among Eastern Romanian Emergency Medicine Personnel during the Russian–Ukrainian Armed Conflict

**DOI:** 10.3390/healthcare10101976

**Published:** 2022-10-09

**Authors:** Anca Hăisan, Cornelia Măirean, Smaranda Ioana Lupuşoru, Cristina Tărniceriu, Diana Cimpoeşu

**Affiliations:** 1 Emergency Department, “Sf. Spiridon” County Clinical Emergency Hospital, 700111 Iaşi, Romania; 2 Department of Surgery, University of Medicine and Pharmacy “Grigore T. Popa”, 700115 Iaşi, Romania; 3 Faculty of Psychology and Educational Sciences, Alexandru Ioan Cuza University, 700554 Iași, Romania; 4 General Surgery Department, “Sf. Spiridon” County Clinical Emergency Hospital, 700111 Iași, Romania; 5 Hematology Clinic, “Sf. Spiridon” County Clinical Emergency Hospital, 700111 Iași, Romania; 6 Department of Morpho-Functional Sciences I, Discipline of Anatomy, University of Medicine and Pharmacy “Grigore T. Popa”, 700115 Iaşi, Romania; 7 Faculty of Medicine, University of Medicine and Pharmacy “Grigore T. Popa”, 700115 Iasi, Romania

**Keywords:** threats, anxiety, health, war, emergency unit

## Abstract

(1) Background: The aim of the current study is to identify the relations between worries, threat perceptions, anxiety states, and general health in a sample of Emergency unit staff during the Russian Ukrainian armed conflict. The sample consisted of 372 Emergency Care staff (M age = 39.41; 77.2% women), physicians, and nurses from North-eastern Romania. (2) Methods: Participants completed an online survey including scale measuring worries, recent anxiety symptoms, and general health. The survey was distributed and completed in the next month after the outbreak of the war. (3) Results: The results showed that the worries, perceived threats, and anxiety symptoms during the last period are negatively related to general health. Women presented higher levels of worries, perceived threats, and anxiety symptoms, compared to men, while for general health, women presented lower scores. Concerning the differences between different professional categories, the results showed that trainees presented higher scores on worries, perceived threats, and anxiety symptoms, as well as lower scores on general health. The implications of these results for improving health and quality of life are discussed. (4) Conclusions: The level of stress increases beyond the borders where the war takes place, thus indirectly affecting the Emergency Care staff involved in the care of victims. Clinical implications of these results for interventions designed to monitor quality of life and to prevent unwanted consequences of exposure to traumatic life events are discussed.

## 1. Introduction

In the aftermath of catastrophes and disasters (e.g., mass shootings, hurricanes, terrorism, floods), people respond with a variety of negative psychological outcomes, including depression, anxiety, traumatic stress, etc. [1,2]. An atypical polytraumatic event for the 21st century started on 24 February 2022, when Ukraine was attacked and invaded by the Russian army. Horrific war scenes, threats to life, loss of life, property, family members and communities, separation from family, injury, illness, and long-term disability are several examples of the atrocities that became known worldwide. In order to protect lives, an exodus of Ukrainian people to safer countries could be observed, mainly to neighboring European countries (e.g., Romania, Poland) that provided shelter, food, medical, and psychological care to refugees. Some statistics reported that over a million refugees from Ukraine crossed the borders only in the first week of war, while other authors maintain that this exodus (including primarily women with children) is unprecedented since the Second World War [3,4].

The border between Romania and Ukraine is an international border, 649.4 km long, which delimits the territories of Romania and Ukraine. It is one of the eastern borders of the European Union since its enlargement in 2007 [5]. On the morning of 24 February 2022, the Russian-Ukrainian armed conflict began. This caused Europe’s largest refugee crisis since World War II [6] and since the beginning of the war, one-third of Ukrainians have been forced from their homes [7]. According to the current data (8 July 2022), there are 8.8M refugee arrivals from Ukraine [8,9]. Romania has registered 786.839 (5 July 2022) border crossings from Ukraine [8]. Most of the refugees are women and children (>90%) and in Romania estimates suggest that 33% are children [10]. Ukrainian refugees entered the North-East and East of Romania by the following border crossings: Siret, Radauti Prut, Stânca, Sculeni, Albița, Oancea, and Galați [11,12]. This influx of refugees caused a high pressure on the government, ONGs, health systems, and on civil society [13], especially at the beginning of the invasion, as the majority of the refugees were and are hosted in private homes [14].

Previous studies on the mental health impacts of war (e.g., civil wars in the former Republic of Yugoslavia) focused on several types of populations, such as children, adults, victims of torture, and refugees. Overall, the results suggested that people exposed to war present higher rates of post-traumatic stress, anxiety, depression, and somatic complaints [3]. Perceived threats generated by armed conflict (health, security, economic, and political threats) were positively related with anxiety manifestations [15]. While the negative effect of trauma on mental health is well documented, little is known about how indirect exposure to the war-affected population is associated with negative psychological manifestations. According to DSM-5 [16], indirect exposure to traumatic life events could lead to the same pattern of results as direct exposure. Indirect victims are represented by people not directly involved in the war conflict, but who interact with direct victims. In countries hosting refugees, the pathological mental condition associated with the trauma could be transmitted in a vicarious way from the direct victims to other people (e.g., volunteers, those who provide health care services, etc.) [3]. Thus, terrible events affect not only people directly involved, but they could also have a significant impact on the mental health of rescue workers, including medical personnel [2,17,18].

Empirical results from studies conducted with nurses and physicians from the emergency departments documented the presence of peritraumatic distress, burnout, anxiety, depression, somatic symptoms, and posttraumatic stress reactions (e.g., intrusions, hyper-arousal) [2,19]. Even if all these professionals are able to use their abilities in order to save lives, they may develop long-term negative psychological traumatic manifestations [20]. As far as we know, no previous study has explored the mental health manifestations (e.g., anxiety symptoms, general health) and related variables (worries, perceived threats) reported by healthcare providers in the context of indirect exposure to war and its atrocities. Thus, in order to fill in a gap in the literature, in the current study we aim to explore the relation between perceived threats, anxiety symptoms, and general health in a sample of emergency staff (e.g., nurses, and physicians from the emergency department), in the context of the Russian–Ukrainian war. We also want to explore the role of different demographic information (i.e., gender, professional experience, profession) in shaping psychological manifestations as response to war exposure. Based on previous studies presented above, we expect to find a negative relation between perceived threats, anxiety symptoms, and the general health.

## 2. Materials and Methods

### 2.1. Participants

The sample of the present study consisted of 372 Emergency Care staff from emergency units from five county hospitals and six city hospitals situated in the North-Eastern region, at the border with Ukraine and Moldova. All other medical care facilities without emergency department were excluded. From all these eleven hospitals, one is the only university center from our region, where the trainees are educated. Out of the total sample, 56.7% are nurses, 16.7% are trainees, 13.7% are consultant physicians, and 12.9% are registrars. Participants’ age is between 22 and 66 years old (M age = 39.41; S = 9.84). The number of years of professional experience vary between less than a year and 5 years, with an average of 10.52 (SD = 8.97). Women largely comprised the sample (77.2%).

### 2.2. Measures

#### 2.2.1. War Worries

Five items were used to assess worries, generated by the war outbreak, about financial instability, personal and family safety, and job. The items were evaluated on a 5-point response scale ranging from 1 = not at all to 5 = very much. A total score was computed by summing the responses. The Alpha Cronbach coefficient is 0.92.

#### 2.2.2. Perceived Threats

According to Marciano et al. (2022) [15], we investigated four perceived threats, with a single item which was phrased similarly containing a difference in the specific threat wording: “How much do you feel threatened these days by the (health/economic/security/political) risk?”. The participants completed the items with the instruction to think about the Russian–Ukrainian war. The 5-point response scales ranged from 1 = “not threatening at all” to 5 = “threatening very much.” Two total scores were computed, by summing the responses. The Alpha Cronbach coefficients are 0.82 for war threat and 0.84 for pandemic threat.

#### 2.2.3. Anxiety Symptoms

Beck Anxiety Inventory [21] is a 21-item scale measuring anxiety symptoms. Respondents are asked to rate how much they have been bothered by the symptoms presented in each item after the Russian-Ukrainian war outbreak, including today. Responses were offered on a four-point scale ranging from 0 (“not at all”) to 3 (“severely—I could barely stand it”). Items are summed and higher scores represent greater levels of anxiousness. In the current sample, Alpha Cronbach coefficient is 0.93.

#### 2.2.4. General Health

The General Health Questionnaire (GHQ-12) [22] consists of 12 statements to which respondents indicate agreement on a four-point scale (0 = “not at all”; 3 = “more than usual”). The total score was computed by summing the items. Higher scores represent higher levels of general health. In the present sample, the Alpha Cronbach coefficient is 0.91.

Demographic variables were collected via a questionnaire that covered age, gender, number of years of professional experience, and professional category.

### 2.3. Procedure

All approximately 500 medical workers in the emergency departments of north-eastern cities of Romania were invited to participate in a study on their perceptions about daily professional life and ways of dealing with day-to-day challenges. The invitation was sent online, to the heads of sections to be distributed to their teams, including the informed consent and the link to the study. They were informed about the fact that participation is voluntary and the information will be kept confidential. A large part from the target population responded to our invitation and completed the survey (N = 372). The survey was completed anonymously after signing the informed consent. The scales were presented in the following order: general health, current threats generated by war, worries generated by war, anxiety symptoms, and demographic variables. Participants completed an online survey in the first month after the war outbreak. The average time for filling in the scales is 15 min.

The study was conducted in accordance with the Declaration of Helsinki, and approved by the Ethics Committee of Saint Spiridon County Hospital Iasi (Approval Code: 23, Approval Date: 11 March 2022). Informed consent was obtained from all subjects involved in the study.

### 2.4. Overview of the Statistical Analysis

Firstly, we conducted the preliminary analyses to assess whether socio-demographic variables (age, participants’ gender, and profession) are related to general health. Then, zero-order correlations among the main study variables were computed. Finally, hierarchical regression analyses were performed in order to predict the participants’ general health, using war worries, threat-perception related to war, and also anxiety state.

## 3. Results

### 3.1. Gender Differences in the Main Study Variables

The independent sample *t*-test revealed significant differences between males and females concerning worries, threat perception related to war, anxiety state, and general health. Men presented lower levels of worries, threat perceptions related to war, anxiety, and also higher levels of general health, compared to women. The other differences between males and females are non-significant. These results are presented in Table 1.

### 3.2. Differences between Professional Categories in the Main Study Variables

In order to assess the differences between different professional categories (i.e., trainees, registrars, consultants, nurses) in terms of worries, threat perceptions, anxiety state, and general health, we used the analysis of variance. The results showed significant differences between professional categories in all the outcome variables. Nurses have lower levels of worries and perceive fewer threats related to war compared to trainee physicians. Further, trainees reported high levels of anxiety compared to nurses and consultants. As for general health, nurses reported high levels compared to trainee and specialist physicians. The results are presented in Table 2.

### 3.3. Associations among the Main Study Variables

Zero-order associations showed that general health is negatively related with worries, threat perceptions related to war, and anxiety symptoms. Moreover, both worries and perceived threats are positively related to anxiety symptoms. The participants’ age was negatively related to threat perceptions related to war and also with anxiety symptoms. Moreover, age is positively related to general health. Number of years of experience in the profession is negatively related to anxiety symptoms. These relations are medium to strong [23]. These results are presented in Table 3.

### 3.4. Regression Analyses

To examine how much variance in participants’ general health is explained by worries, threat perception, and anxiety, we conducted a hierarchical multiple regression analysis in which general health was the dependent variable. Age, profession, and gender were included in the analyses, in Step 1. Worries, threat perception, and anxiety state were entered during Step 2.

Demographic variables (i.e., age, profession, gender), worries, threat perception, and anxiety symptoms explained a 52% variance in general health (see Table 4). Anxiety state is a significant negative predictor. Threats related to war and worries did not predicte self-reported general health.

## 4. Discussion

The aim of the current study is to assess the predictors of general health in a sample of Emergency Care staff during the Russian–Ukrainian armed conflict.

The results showed that participants that reported high levels of anxiety symptoms after the war outbreak also reported low level of general health. Therefore, anxiety state should be targeted in interventions designed to monitor quality of life and to prevent unwanted consequences of professional exposure to traumatic life events.

Women presented higher levels of worries, perceived threats, and anxiety symptoms, compared to men, while for general health, women presented lower scores. Concerning the differences between different professional categories, the results showed that trainees presented higher scores for worries, perceived threats, and anxiety symptoms, as well as lower scores for general health. In a previous study conducted with Romanian physicians from Emergency Care staff [19], trainees or senior house officers represent the professional category with a high probability of developing burnout manifestations. These results are in line with the fact that, in the present study, more years of professional experience were associated with low anxiety symptoms. Therefore, as a result of cumulative exposure, people may develop coping strategies that help them to adapt to different professional challenges. Based on these results, young people with less experience in the medical field should be particularly targeted for intervention programs, in order to help them to identify coping mechanism with an adaptative role in difficult situations.

In a world report published in 2022, Lancet [24] showed that “‘Today in Ukraine, medics are on the front line, working tirelessly to save lives while risking their own…”, but at the same time, the level of stress is much higher than usual among the Emergency Care staff working in the emergency units at the borders of the armed conflict. The medical staff from the emergency units from the eastern border of Romania treats refugee patients, either for acute medical illnesses or trauma, or for imbalances of chronic diseases. In addition to medical care, emergency workers also identify the drama of refugee patients from Ukraine. Thus, many people in this field volunteered at the country’s borders to provide emergency medical care. The biggest wave of refugees entered Romania during a period of abnormally cold weather, with rain and snow. The most affected by these unfavorable weather conditions were children and the elderly, these being also the category of people who needed special attention. This increased vulnerability of these categories of people has led to a greater involvement of Emergency Care staff which has led to a higher risk of burn out.

All these aspects show the impact of the armed conflict at a distance from the place of its occurrence. Several recent studies have objectively shown the impact of disasters on the mental health of victims but also on those involved in rescuing victims [1,2,15]. Continuing this idea of the impact of armed conflict, our study brings objective data that show that the level of stress increases beyond the borders where the war takes place, indirectly affecting the medical staff involved in the care of victims.

A recent published work showed that European countries may experience public health threats due to the influx of war refugees [4]. The authors described threats and challenges to public health related to infectious diseases. In this context, we would like to mention that the Russian–Ukrainian armed conflict came immediately at the end of the Covid 19 pandemic, which corresponds to an already tired, stressed, and overworked medical personnel as a result of the pandemic. The main challenge for the health system of these countries in this situation is how to develop adaptation measures to provide medical care to both residents of their own countries and refugees, and to protect the own medical personnel against burn out. In order to provide optimal care for those in need, additional effort is needed to identify at-risk individuals for mental health impairments. Therefore, screening protocols should be used in order to identify people more affected by the cost of caring, that should be targeted in further interventions. Trainings and intervention designed to reduce anxiety and to process difficult experiences, like Critical Incidents Stress Debriefing, implemented shortly after critical events or periods, could be addressed. Thee interventions could be useful not only for venting negative emotions and for facilitating adjustment to critical situations, but also to prevent long-term, unwanted consequences for professional and personal quality of life. Providing emotional support and psychoeducation focusing on teaching skills and functional coping mechanisms with intense stress could be the key elements for any intervention designed to increase resilience in the face of critical life events.

Several limitations of the present study should be noted. Firstly, the design of the present study is cross-sectional, which impedes us to sustain the existence of causal relations between variables. Future longitudinal studies, with different waves, are needed in order to identify how anxiety states and worries about particular events affect the overall level of health. Secondly, we used self-report measures that can be affected by subjectivity biases. Particularly for general health, self-report measures could be combined with some objective measures, in order to obtain a more complex picture of overall personal health. Third, the fact that our sample is mainly composed by women could limit the generalizability of our results. This limitation could be due to the fact that women are more willing to be volunteers in research or are more willing to admit and report their difficulties in terms of mental health. Future studies with samples comprising both women and men are needed in order to better understand the particularities of general health during challenging times in Emergency Care staff.

## 5. Conclusions

Medical personnel from the emergency units in the North-East region of Romania reported high levels of anxiety symptoms after the Russian–Ukrainian war outbreak and also reported low levels of general health. Therefore, anxiety state should be targeted in interventions designed to monitor quality of life and to prevent unwanted consequences of professional exposure to traumatic life events. The level of stress increases beyond the borders where the war takes place, thus indirectly affecting the Emergency Care staff involved in the care of victims.

## Figures and Tables

**Table 1:** Gender differences in the main study variables.

Variables	Male	Female		
	*M* (*SD*)	*M* (*SD*)	*t*-Value	*p*-Value
Worries	16.08 (5.48)	18.44 (4.98)	−3.75	<0.001
Threat_war	11.08 (3.27)	12.13 (2.97)	−2.79	0.006
Anxiety	28.98 (9.93)	33.75 (10.58)	−3.66	<0.001
General health	36.61 (5.99)	34.82 (6.57)	2.26	0.024

**Table 2:** Differences between professional categories in the main study variables.

Variables	Consultants	Registrars	Trainee	Nurses		
	*M* (*SD*)	*M* (*SD*)	*M* (*SD*)	*M* (*SD*)	*F*	*p*-Values
Worries	18.47 (4.92)	18.83 (4.96)	19.87 (4.03)	16.98 (5.40)	6.1	<0.001
Threat_war	12.43 (2.62)	12.47 (2.86)	13.14 (1.99)	11.26 (3.32)	7.93	<0.001
Anxiety	30.82 (9.26)	34.41 (10.57)	37.93 (9.70)	31.11 (10.69)	7.79	<0.001
General health	34.94 (6.97)	32.45 (6.13)	32.16 (6.82)	36.81 (5.80)	12.88	<0.001

**Table 3:** Zero-order correlations among the main study variables.

	1.	2.	3.	4.	5.	6.	7.
1. Worries							
2. Threat_war	0.82 ***						
3. Anxiety	0.63 ***	0.53 ***					
4. General health	−0.47 ***	−0.43 ***	−0.70 ***				
5. Age	−0.08	−0.10 *	−0.19 ***	0.10 *			
6. Experience	−0.06	−0.09	−0.14 **	0.05	0.70 ***		
7. Gender	0.19 ***	0.14 **	0.19 ***	−0.11 *	−0.04	−0.11 *	
Mean	17.90	11.89	32.65	35.21	39.41	10.52	
SD	5.18	3.70	10.61	6.48	9.84	8.97	

Note: * *p* < 0.05, ** *p* < 0.01, *** *p* < 0.001.

**Table 4:** Hierarchical linear regression analysis examining the role of worries, threats, and anxiety in prediction of general health.

Variables		General Health		
	*B*	*SE*	*β*	*p*
Step 1				
Age	0.08	0.03	0.13	0.012
Profession	−1.28	0.30	−0.21	<0.001
Gender	−1.91	0.79	−0.12	0.016
*R* ^2^		0.07 *		
Step 2				
Age	−0.008	0.02	−0.01	0.781
Profession	−0.89	0.22	−0.15	<0.001
Gender	0.10	0.58	0.007	0.855
Worries	0.08	0.09	0.06	0.266
Threat_war	−0.20	0.13	−0.09	0.103
Anxiety	−0.42	0.03	−0.69	<0.001
*R* ^2^		0.52 **		
*R*^2^ch		0.45 **		

Note: * *p* < 0.01, ** *p* < 0.001.

## Data Availability

The data that support the findings of this study are openly available in https://zenodo.org/referencenumber10.5281/zenodo.7153852.
